# High Glucose-enhanced Acetylcholine Stimulated CGMP Masks Impaired Vascular Reactivity in Tail Arteries from Short-Term Hyperglycemic Rats

**DOI:** 10.1155/EDR.2000.69

**Published:** 2000

**Authors:** Marwan Hamaty, Cristina B. Guzmán, Mary F. Walsh, Ann M. Bode, Joseph Levy, James R. Sowers

**Affiliations:** 1 Division of Endocrinology Diabetes and Hypertension Wayne State University School of Medicine and VAMCI Detroit MI USA; 2 Department of Exercise and Movement Science University of Oregon Eugene OR USA; 3 Division of Endocrinology Diabetes and Hypertension SUNY HSC at Brooklyn 450 Clarkson Avenue, Box 1205 Brooklyn NY 11203 USA

## Abstract

Impaired vascular endothelium-dependent relaxation and augmented contractile responses have been reported in several models of long-term hyperglycemia. However, the effects of short-term ambient hyperglycemia are poorly understood. Since oxidative stress has been implicated as a contributor to impaired vascular function, we investigated the following:

Aims: (1) the effects of high glucose exposure *in vitro* (7 – 10 days) on vascular relaxation to acetylcholine (Ach) and contractility to norepinephrine (NE) and KCl; (2) if NO-dependent cGMP generation is affected under these conditions; and (3) aortic redox status.

Methods: Non-diabetic rat tail artery rings were incubated in normal (5mM) (control NG) or high (20mM) glucose buffer (control HG). Vascular responses to Ach, NE and KCl were compared to those of streptozotocin (SZ) diabetic animals in the same buffers (diabetic NG, diabetic HG). Ach stimulated cGMP levels were quantitated as an indirect assessment of endothelial nitric oxide (NO) production and oxidative stress evaluated by measuring vascular glutathione and oxidized glutathione.

Results: Rings from diabetic rats in NG showed impaired relaxation to Ach (*P* = 0.002) but relaxed normally, when maintained in HG. Similarly, contractile responses to NE were attenuated in diabetic rings in NG but similar to controls in HG. HG markedly augmented maximal contraction to KCl compared to control and diabetic vessels in NG (*P* < 0.0001). Diabetic vessels in a hyperosmolar, but normoglycemic, milieu respond like those in HG. *in vitro*, HG for 2 hours changed neither relaxation nor contractile responses to NE and KCl in control rings. Basal cGMP levels were lower in aortae from diabetic animals pre-incubated in NG than in HG/LG or in control rings in NG (*P* < 0.05). cGMP responses to Ach were exaggerated in diabetic vessels in HG (*P* = 0.035 *vs*. control NG, *P* = 0.043 *vs*. diabetic NG) but not different between control and diabetic rings in NG. Vessels from diabetic animals had lower levels of GISH (*P* < 0.0001) and higher levels of GSSG (*P* < 0.0001) indicating oxidative stress.

Conclusions: Our data indicate that endothelium dependent relaxation is altered early in the diabetic state and that increased NO responses may compensate for augmented oxidative stress but the lack of effect of short-term exposure of normal vessels to HG suggests that short-term hyperglycemia *per se* does not cause abnormal vascular responses.


*Int. Jnl. Experimental Diab. Res.*, Vol. 1, pp. 69–79
Reprints available directly from the publisher
Photocopying permitted by license only

© 2000 OPA (Overseas Publishers Association) N.V.
Published by license under
the Harwood Academic Publishers imprint,
part of The Gordon and Breach Publishing Group.
Printed in Malaysia.

### High Glucose-enhanced Acetylcholine Stimulated CGMP Masks Impaired Vascular Reactivity in Tail Arteries from Short-term Hyperglycemic Rats

MARWAN HAMATY[a], CRISTINA B. GUZMÁN[a], MARY F. WALSH[a], ANN M. BODE[b], JOSEPH LEVY[a] and JAMES R. SOWERS[c,*]

[a] *Division of Endocrinology, Diabetes and Hypertension, Wayne State University School of Medicine and VAMCI, Detroit, MI ;* [b] *Department of Exercise and Movement Science, University of Oregon, Eugene, OR;* [c] *Division of Endocrinology, Diabetes and Hypertension, SUNY HSC at Brooklyn, Brooklyn, NY*

*(Received 16 May 1999; Revised 14 October 1999; In final form 15 October 1999)*

**Impaired vascular endothelium-dependent relaxation and augmented contractile responses have been reported in several models of long-term hyperglycemia. However, the effects of short-term ambient hyperglycemia are poorly understood. Since oxidative stress has been implicated as a contributor to impaired vascular function, we investigated the following:**
**Aims: (1) the effects of high glucose exposure *in vitro* (7–10 days) on vascular relaxation to acetylcholine (Ach) and contractility to norepinephrine (NE) and KCl; (2) if NO-dependent cGMP generation is affected under these conditions; and (3) aortic redox status.**
**Methods: Non-diabetic rat tail artery rings were incubated in normal (5 mM) (control NG) or high (20 mM) glucose buffer (control HG). Vascular responses to Ach, NE and KCl were compared to those of streptozotocin (SZ) diabetic animals in the same buffers (diabetic NG, diabetic HG). Ach-stimulated cGMP levels were quantitated as an indirect assessment of endothelial nitric oxide (NO) production and oxidative stress evaluated by measuring vascular glutathione and oxidized glutathione.**
**Results: Rings from diabetic rats in NG showed impaired relaxation to Ach ($P = 0.002$) but relaxed normally, when maintained in HG. Similarly, contractile responses to NE were attenuated in diabetic rings in NG but similar to controls in HG. HG markedly augmented maximal contraction to KCl compared to control and diabetic vessels in NG ($P < 0.0001$). Diabetic vessels in a hyperosmolar, but normoglycemic, milieu respond like those in HG. *In vitro*, HG for 2 hours changed neither relaxation nor contractile responses to NE and KCl in control rings. Basal cGMP levels were lower in aortae from diabetic animals pre-incubated in NG than in HG/LG or in control rings in NG ($P < 0.05$). cGMP responses to Ach were exaggerated in diabetic vessels in HG ($P = 0.035$ *vs.* control NG, $P = 0.043$ *vs.* diabetic NG) but not different between control and diabetic rings in NG. Vessels from diabetic animals had lower levels of GISH ($P < 0.0001$) and higher levels of GSSG ($P < 0.0001$) indicating oxidative stress.**
**Conclusions: Our data indicate that endothelium-dependent relaxation is altered early in the diabetic state and that increased NO responses may compensate for augmented oxidative stress but the lack**

*Corresponding author. Director, Division of Endocrinology, Diabetes and Hypertension, SUNY HSC at Brooklyn, 450 Clarkson Avenue, Box 1205, Brooklyn, NY 11203. Tel.: 718 245-5020, Fax: 718 270-1699, e-mail: bklynendo@aol.com

**of effect of short-term exposure of normal vessels to HG suggests that short-term hyperglycemia *per se* does not cause abnormal vascular responses.**

*Keywords:* Hyperglycemia, cyclic GMP, endothelium, vascular, streptozocin, rats

### INTRODUCTION

Hyperglycemia-induced alterations in vascular endothelium and/or vascular smooth muscle (VSM) metabolism are thought to mediate many of the changes in vascular tone seen in both human (Johnstone *et al.*, 1993) and animal diabetic states (Kamata *et al.*, 1989; Durante *et al.*, 1988; Graier *et al.*, 1993, 1996). However, current information on vascular tone in diabetes is largely derived from long-term animal models where irreversible arterial wall changes such as glycation and collagen deposition (Ruderman *et al.*, 1992) may contribute to endothelial and/ or VSM dysfunction (Poston and Taylor, 1995). Indeed, in human disease, impairment of endothelium-dependent relaxation does not correlate with glycemic control (Johnstone *et al.*, 1993) and normal vascular relaxation to acetylcholine (Ach) has been reported in uncomplicated type 2 diabetes mellitus (Avogaro *et al.*, 1997). Also, in short-term hyperglycemia (1 and 7 hrs), contractile responses to norepinephrine (NE) were unaffected in normal subjects using forearm perfusion techniques (Houben *et al.*, 1993, 1994). In animal models, responses appear to vary in different vascular beds; changes in endothelial and/or vascular smooth muscle NO production are thought to mediate, at least in part, the high glucose effects on vascular reactivity (Bohlen and Lash, 1993; Taylor and Poston, 1994; Wascher *et al.*, 1995; Cipolla *et al.*, 1997). High glucose concentrations have been reported to decrease NO production by some investigators (Gupta *et al.*, 1992) but, others have shown that high glucose concentrations enhance NO production perhaps as a compensatory mechanism for the associated excess superoxide radicals and oxidative stress (Graier *et al.*, 1996).

This investigation was conducted to examine endothelial mediated relaxation to Ach and contractile responses to NE and potassium chloride (KCl) in the tail artery of short-term (7–10 days) diabetic rats before physical glucose-dependent changes of the arterial wall occur since glucose-induced glycation and/or collagen deposition may enhance arterial wall stiffness and lead to erroneous conclusions regarding endothelium-dependent effects. Thus, contractile properties of rings from both control and diabetic animals incubated *in vitro* in high glucose (HG) were compared to those incubated in normal glucose buffer (NG). These experiments were designed to simulate *in vivo* hyperglycemia and to test responses of diabetic vessels when changed from a high glucose milieu to a normal glucose environment. L-glucose was used to control for osmolar effect(s). Ach-stimulated cGMP levels were measured as a surrogate for vascular NO generation. Measurement of antioxidants such as ascorbic acid and glutathione were carried out to test whether changes of cGMP levels are associated with oxidative stress.

### METHODS

### Animals

The institutional Animal Investigations Committee approved all procedures in this study. Male Sprague-Dawley rats (200–250 gm) from Charles River (Wilmington, MA) were housed two per cage and given free access to rat chow and water. Diabetes mellitus was induced by a single intraperitoneal injection of streptozotocin (SZ 65 mg/kg, Sigma, St. Louis. MO). Hyperglycemia was documented by monitoring tail vein blood glucose. Under anesthesia (pentobarbital 65 mg/kg), animals were sacrificed after 7–10 days of hyperglycemia by inducing pneumothorax and blood samples were obtained for serum glucose measurement (Yellow Springs Instruments, Yellow Springs, OH). Age-matched rats were used as controls.

### Vascular Reactivity

Tail arteries were dissected free from connective tissue and placed in ice-chilled normal buffer containing $MgSO_4$ 1.20, NaCl 125, KCl 4.70, $KH_2PO_4$ 1.20, $CaCl_2$, $2H_2O$ 1.60, $NaHCO_3$ 84.01, Dextrose 5 and NaEDTA 0.03 mM. Arteries from diabetic animals were divided into two groups: one placed in normal buffer (diabetic NG) and another kept in high glucose buffer (diabetic HG) containing 20 mM D-glucose with the same ionic composition as normal buffer. A buffer containing 5 mM D-glucose + 15 Mm L-glucose (diabetic LG) was employed as osmotic control. We chose L-glucose because it enters the cell in a manner similar to D-glucose but does not undergo any further metabolism, thus avoiding cell shrinkage (Ibu and Short, 1986). Three millimeter rings were suspended from isometric force transducers (Gould instrument, Cleveland, OH) in muscle baths containing the buffers described above at 37°C and aerated with a mixture of 95% $O_2$ and 5% $CO_2$, pH 7.4. Rings were stretched to 1 gm tension and allowed to stabilize for 30 min before adding agonists (Walsh *et al.*, 1996). The presence of intact endothelium was confirmed in each ring by demonstrating normal relaxation to Ach ($10^{-5}$ M) after submaximal contraction with KCl. Diabetic NG rings were placed in buffer 2 hours before starting the experiment. In order to investigate the effects of high glucose *in vitro*, agonist dose responses were performed on arteries from control animals before and after 2-hour incubation with high glucose buffer (control HG). Responses studied included relaxation to cumulative doses of Ach ($10^{-12}$–$10^{-5}$ M) after submaximal contraction with KCl (35 mM, 80–90% of maximal contractility), contraction to NE ($10^{-10}$–$10^{-5}$ M) and to KCL (10–45 mM).

### cGMP Assay

Aortae were used for measuring cGMP because they provide a larger sample and respond similarly to tail vasculature (Walsh *et al.*, 1996). Experiments were performed in conditions similar to those for vascular reactivity (control NG, control HG, diabetic NG, diabetic HG and diabetic LG). The phosphodiesterase inhibitor IBMX (1 mM) was added to the medium 30 min before obtaining basal samples. Ach was then added at a dose of $10^{-5}$ mM and the reaction terminated at 30, 120 and 300 sec. To test if changes in basal and/or stimulated cGMP levels were NO mediated, these experiments were repeated in the presence of L-NAME (0.1 mM for 30 min). All samples were snap frozen using dry ice, homogenized and ether extracted on the day of cGMP assay *via* ELISA (Cayman, Ann Arbor, MI). Values were adjusted for the amount of protein in the samples (Biorad, Hercules, PA).

### Glutathione and Gllutathione Disulfide Levels

GSH and GSSG were measured by high-performance liquid chromatography (HPLC) according to Bode (Rose and Bode, 1995). Samples were sonicated in 1% picric acid, centrifuged and the supernatant fractions assayed for GSH or GSSG.

### Statistical Analysis

Two-way analysis with Fisher's protected least significant difference and repeated measure ANOVA were used to compare contractility dose response curves when expressed as absolute values (mg tension) and to compare Ach-stimulated cGMP responses among vessels groups. One-way ANOVA with Neuman–Keuls multiple comparison test was used to study cGMP responses to Ach in each group and to compare basal cGMP levels among groups. $ED_{50}$ values for dose responses curves were calculated using the Pharm C Program (Springer-Verlag, New York, NY) and differences were determined by Student's t-test, Differences in body weight and serum glucose and GSH/GSS levels were also evaluated using Student's t-test, Values in the text are given as mean ± SEM and $P < 0.05$ is considered significant.

### RESULTS

### Animal Profile

Short-term diabetes did not significantly affect body weights (291 ± 13 in diabetic animals *vs.* 283 ± 11 gm in control) but serum glucose levels were higher in the SZ injected animals (32.6 ± 0.9 *vs.* 11.9 ± 0.9 mM, $P < 0.0001$).

### Vascular Reactivity

Responses to NE and KCl: two-hour *in vitro* incubation of tail artery rings with high glucose buffer failed to alter contractile responses to either NE or KCl (data not shown). Maximal responses to NE were similar in all groups (Tab. I). Responses in rings from diabetic animals incubated in NG were attenuated at lower concentrations of NE ($10^{-9}$ M to $10^{-7}$ M) when compared to diabetic vessels maintained in HG or control vessels in NG. This effect was significant only between rings of diabetic animals in HG and diabetic in NG ($P$=0.014) when calculated over the entire curve. However, sensitivity in these vessels was significantly decreased, $ED_{50}$ was doubled, compared with those kept in HG and control vessels in NG (Tab. I) ($P$=0.005 for control NG *vs.* diabetic NG, $P$=0.0003 for diabetic HG *vs.* diabetic NG) (Fig. 1). Responses to KCl were higher in diabetic tail artery rings in HG when compared to control in NG (maximal contraction: control NG 967 ± 108 *vs.* 1310 ± 117 mg tension in diabetic HG, $P < 0.0001$) with no change in sensitivity. In contrast, responses to KCl in diabetic rings maintained in NG for 2 hours were identical to control (maximal contraction for diabetic NG 981 ± 73.7 mg tension) (Fig. 2).

### Relaxation to Ach

Preincubation in 20 mM glucose for 2 hrs did not change KCl-induced submaximal contraction (748 ± 87 *vs.* 708 ± 83 mg in 5 mM glucose). Maximal relaxation to Ach was also similar in both groups (control NG 308 ± 41 *vs.* 320 ± 46 mg tension in control HG). As seen in Figure 3A, relaxation curves were significantly different ($P$ = 0.003 by two-way ANOVA). Rings exposed to HG concentration relaxed to a greater extent at low doses of Ach but sensitivity remained unimpaired ($ED_{50}$: control NG $13.6 \pm 4.1 \times 10^{-8}$ *vs.* $12.9 \pm 6.9 \times 10^{-8}$ M in control HG, $ED_{16}$: control NG $34 \pm 8.7 \times 10^{-10}$ *vs.* $25.9 \pm 11.3 \times 10^{-10}$ M in control HG). Vessels from streptozotocin treated rats maintained in high glucose buffer (HG) relaxed to Ach to the same extent as those of control vessels in normal glucose buffer (NG). On the other hand, in NG, maximal relaxation remained similar to control but sensitivity to Ach was decreased ($P$=0.002 for control NG *vs.* diabetic NG, $P$=0.0009 for diabetic HG *vs.* diabetic NG) (Fig. 3B, Tab. I).

Diabetic rat vessel reésponses to NE, KCl and Ach did not differ in hyperosmolar conditions

TABLE I Relaxation and contractile responses in rat tail artery rings of short-term diabetic rats

| | Control NG | Diabetic NG | Diabetic HG |
|---|---|---|---|
| Submaximal contraction to KCl (mg) | 873 ± 86 | 792 ± 46 | 918 ± 101 |
| Maximal relaxation to Ach (mg) | 298 ± 64 | 217 ± 46 | 278 ± 39 |
| $ED_{50}$(M) | $9.68 \pm 1.1 \times 10^{-8}$ | $26.5 \pm 4.4 \times 10^{-8}$* | $10.5 \pm 2.2 \times 10^{-8}$ |
| Maximal contraction to NE(mg) | 1300 ± 138 | 1521 ± 138 | 1420 ± 109 |
| $ED_{50}$ (M) | $7.6 \pm 1.6 \times 10^{-8}$ | $14.3 \pm 1.0 \times 10^{-8}$** | $6.15 \pm 0.9 \times 10^{-8}$ |

* $P = 0.002$ *vs.* Control NG; $P = 0.009$ *vs.* Diabetic HG.
** $P = 0.005$ *vs.* Control NG; $P = 0.0003$ *vs.* Diabetic HG.

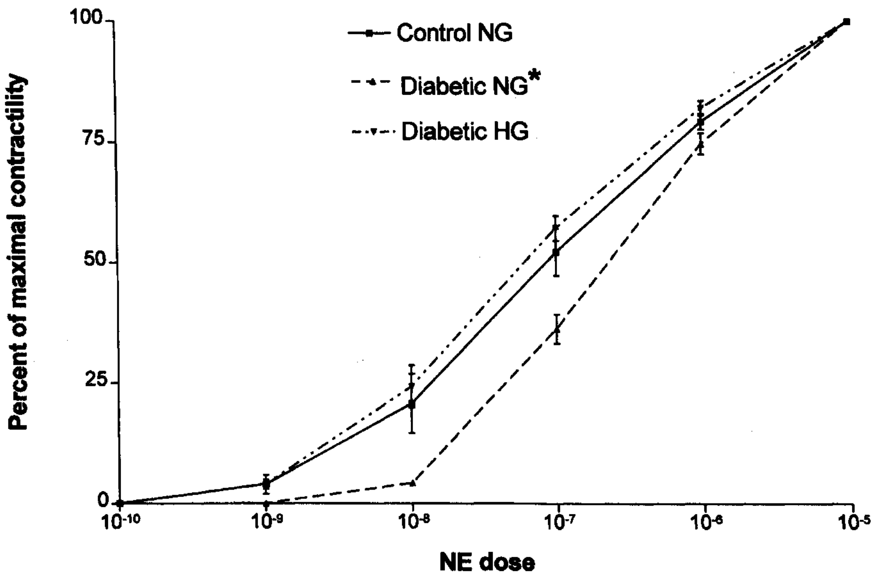

FIGURE 1 Contractile responses to NE: Data are plotted as percent of maximal contraction. Attenuated responses in diabetic rings in NG compared with control in NG and diabetic HG with a significant change in $ED_{50}$ ($P = 0.005$, 0.0003, respectively) but no difference between control in NG and diabetic in HG (control NG, $n = 5$; diabetic NG, $n = 7$; diabetic HG, $n = 5$).

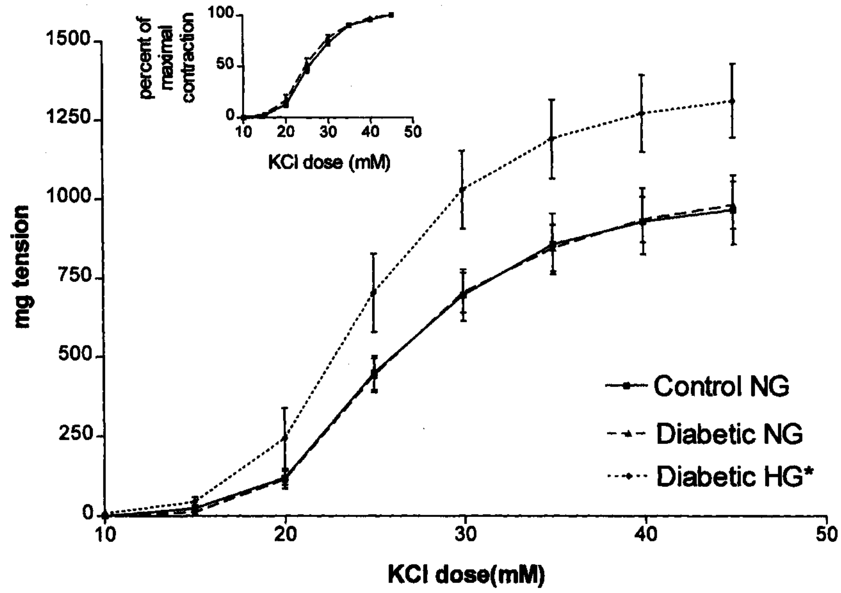

FIGURE 2 Maximal contractility to KCl is markedly enhanced in diabetic rings in HG compared with control vessels in NG ($P < 0.0001$) while responses in diabetic in NG are identical to control in NG. Sensitivity to KCl is unchanged in diabetic vessels in HG (inset) (control NG, $n = 6$; diabetic NG, $n = 7$; diabetic HG, $n = 5$).

(diabetic LG) from those seen in high D-glucose (Tab. II) suggesting osmolality contributes to these responses.

### Aortic cGMP

Basal cGMP levels in endothelium-intact aortic segments were lower only in vessels from

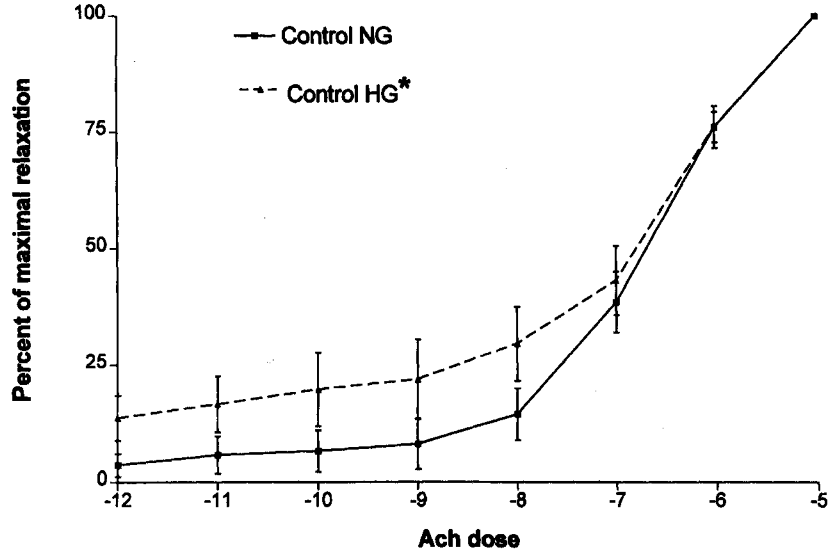

FIGURE 3A Relaxation to Ach after submaximal contraction with KCl in control rings in NG and HG: $P = 0.003$ by two-way ANOVA, no difference in $ED_{50}$ or $ED_{16}$ (control NG $n = 9$, control HG $n = 9$).

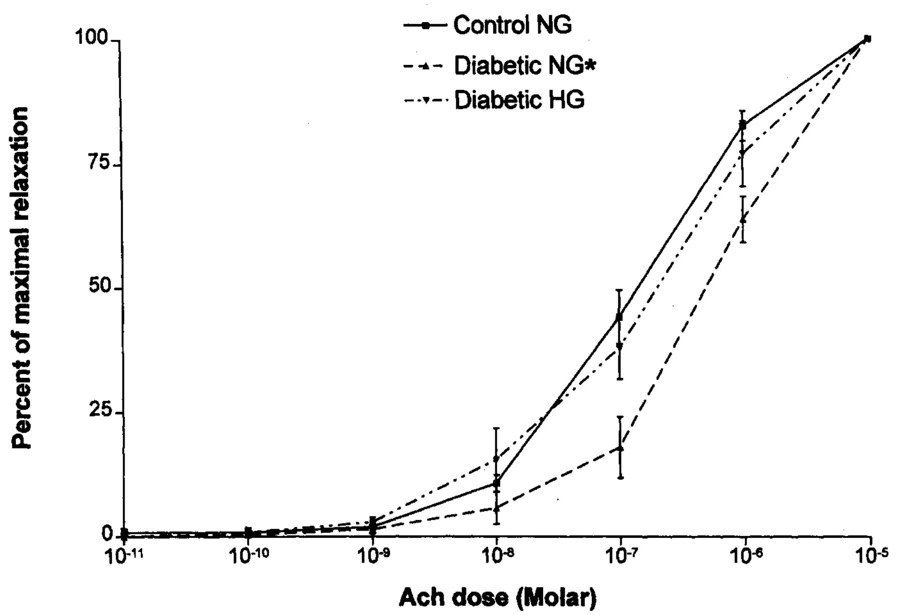

FIGURE 3B Relaxation to Ach after submaximal contraction with KCl. Attenuated relaxation in diabetic vessels in NG with a significant difference in $ED_{50}$ are seen when compared with control in NG and diabetic in HG ($P = 0.002$, $P = 0.009$ respectively) while responses in control in NG and diabetic in HG are similar (control NG, $n = 9$; diabetic NG, $n = 8$ diabetic HG, $n = 7$).

diabetic animals pre-incubated in NG when compared with control in NG and diabetic rings in high D-glucose or L-glucose buffer (Tab. III). Ach effects on cGMP levels in control rings in both NG and HG solutions were not significant, but Ach caused a significant increase in cGMP

TABLE II Relaxation and contractile responses in rat tail artery rings maintained in high D-glucose environment *vs.* high L-glucose environment

| | Diabetic HG | Diabetic LG |
|---|---|---|
| Submaximal contraction to KCl | 873 ± 79 mg | 722 ± 128 mg |
| Maximal relaxation to Ach | 195 ± 53 mg | 267 ± 128 mg |
| $ED_{50}$ | $23.7 \pm 2.2 \times 10^{-8}$ M | $20.5 \pm 2.6 \times 10^{-8}$ M |
| Maximal contraction to NE | 1487 ± 155 mg | 1562 ± 134 mg |
| $ED_{50}$ | $11.69 \pm 3.7 \times 10^{-8}$ M | $9.7 \pm 2.4 \times 10^{-8}$ M |

TABLE III Ach-stimulated cGMP responses in the aorta

| | Basal cGMP | 30 sec | 120 sec | 300 sec |
|---|---|---|---|---|
| Control NG | 12.8 ± 2.2 | 15.8 ± 3.2 | 13.6 ± 5.0 | 16.2 ± 5.5 |
| Control HG | 7.1 ± 2.7 | 32.6 ± 10.2 | 36.2 ± 8.8 | 25.9 ± 8.7 |
| Diabetic NG | 2.8 ± 0.27* | 9.3 ± 4.0 | 29.5 ± 10.8** | 21.7 ± 6.8 |
| Diabetic HG | 14.4 ± 3.0 | 17.7 ± 5.7 | 42.9 ± 9.3** | 32.5 ± 2.6 |
| Diabetic LG | 16.2 ± 2.9 | 45.1 ± 8.3 | 68.1 ± 17.9# | 70.5 ± 12.6# |

* $P < 0.05$ compared with basal cGMP of control NG, diabetic HG and diabetic LG.
** $P < 0.05$ compared with basal cGMP.
\# $P < 0.01$ compared with basal cGMP.

levels compared with baseline after 2 min of exposure to the drug in all three groups of vessels from diabetic animal, *i.e.*, in NG, HG and LG. In the latter, the increase lasted up to 5 minutes (Tab. III). Thus, short-term diabetes resulted in enhanced cGMP responses to Ach while hyprosmolality caused a combination of enhancement and prolongation of the effect. When Ach-stimulated cGMP levels were compared among the groups, no difference between control NG and diabetic rings in NG was seen but continued exposure to high glucose (diabetic HG) exaggerated the responses ($P$=0.02 *vs.* control NG, $P$=0.043 *vs.* diabetic NG). Incubation in a hyperosmolar buffer resulted in a greater increase in cGMP in diabetic rings compared with diabetic vessels kept in HG ($P$=0.0006) and control vessels in NG ($P < 0.0001$). These data suggest that the D-glucose effects on Ach-stimulated cGMP responses are not solely dependent on its osmotic properties. When control rings were pre-incubated in HG, Ach-stimulated cGMP was increased but the difference did not reach statistical significance ($P$=0.059, two-way ANOVA). These Ach-stimulated cGMP responses were mediated by NO release since they were completely abolished by L-NAME (data not shown).

### Redox Status

These experiments were done on aortae from control and diabetic animals only ($n$=10 for each) because only arteries from diabetic animals exhibited altered vascular reactivity and cGMP responses. Samples were frozen immediately after harvesting without any *in vitro* manipulation of glucose concentration. Diabetic animals had strikingly lower glutathione (GSH, control 0.7 ± 0.2 *vs.* 0.38 ± 0.03 in diabetics $P < 0.0001$) and higher oxidized glutahione (GSSG, control 0.15 ± 0.04 *vs.* 0.17 ± 0.02 in diabetics, $P < 0.0001$) indicative of augmented oxidative stress.

### DISCUSSION

Our data show altered endothelium-dependent function in early experimental insulinopenic diabetes. Impairment of vascular relaxation was evident when glucose concentrations were reduced to an artificially normal value *in vitro.*

This impaired relaxation occurred despite a compensatory increase in Ach-elicited cGMP generation that brought cGMP to levels higher than those seen in control rings. These data suggest that impaired vascular relaxation to Ach in diabetic rings in NG is caused by a mechanism distal to and/or independent from NO-mediated cGMP generation. High glucose concentrations (both D-glucose and L-glucose) caused exaggerated cGMP responses to Ach under these conditions. Interestingly, cGMP responses to Ach in vessels from diabetic rats maintained in a hyperosmolar buffer (L-glucose) were much higher than those generated by vessels maintained in a high D-glucose environment. This suggests that D-glucose effects on Ach-stimulated cGMP are the net of multiple factors: hyperosmolality may positively affect cGMP accumulation while glucose metabolism and resultant products may attenuate the osmolar effect of glucose.

Hyperglycemia-mediated alterations in cGMP responses to Ach may reflect changes in NO production, action, free radical/NO scavenging capacity and/or metabolism of cGMP. In results similar to our own, Abiru *et al.*, reported a shift to the right in the concentration-response relaxation curve to Ach in mesenteric arteries of diabetic rats. This was accompanied by lower levels of both cAMP and cGMP (Abiru *et al.*, 1993). However, Graier *et al.*, reported that exposure to high glucose concentrations for 24 hours enhances bradykinin-induced-NO production in cultured aortic endothelial cells (Graier *et al.*, 1996) as well as cGMP generation (Graier *et al.*, 1993) in association with greater concentrations of superoxide radicals (Graier *et al.*, 1996). In our investigation, blockade of NO formation by L-NAME abolished cGMP responses to Ach, supporting the conclusion that differences in cGMP responses to Ach reflect altered NO metabolism. However, we observed augmented cGMP accumulation only in vessels from diabetic animals while the increase in cGMP responses in vessels from control rats placed in high glucose buffer was marginal and did not reach statistical significance. The difference between our results and those cited above may reflect differences in vascular beds, the short period (2 hrs) of high glucose exposure of control vessels in our study, and possibly, a greater ability of phosphodiesterase inhibitors to prevent cGMP degradation in cultured cells in contrast to vascular rings. Our results also show greater oxidative stress in diabetic animals as manifested by reduced glutathione (GSH) and elevated oxidized glutathione (GSSG) content in the vessels. These data are in agreement with Graier's report of a compensatory increase in NO production in response to oxidative stress. Although the effect(s) of glucose on cGMP phosphodiesterase has not been examined in the vasculature, in other cells, glucose and hyperosmolality inhibit cGMP phosphodiesterase activity and enhance cGMP levels (Oyama, 1996; Kuwayama *et al.*, 1996).

Relaxation to Ach in diabetic vessels kept in HG was not commensurate with the higher levels of cGMP. This suggests that enhanced Ach-stimulated cGMP generation compensates for a defect distal to and/or independent from NO-induced cGMP generation. Indeed, the capacity of nitro-compounds to raise cGMP correlates well with their potency to relax contracted vessels (Popescu *et al.*, 1985). Increased production of constrictive prostaglandins (Tesfamariam *et al.*, 1990) and/or superoxide radical (Graier *et al.*, 1996) and decreased generation of endothelium-dependent hyperpolarization factor (Fukao *et al.*, 1997) are other possibilities. Greater oxidative stress in vessels from diabetic animals supports the middle possibility. To date, most *in vitro* contractile/relaxation studies of vessels from diabetic animals have been performed in normal glucose buffer. Of those that were not, and in concert with results obtained in our study, a high *in vitro* glucose milieu ameliorated the impaired sensitivity to Ach in aortae from rats rendered diabetic for 8 weeks (Pieper *et al.*, 1997). Although others have reported no improvement with up to 40 mM glucose (Archibald *et al.*, 1996), these data underscore the importance of maintaining a high glucose or comparable osmolar environment during evaluation of vessels from diabetic animals. Graier *et al.* (1993, 1996) did not observe any L-glucose effect on either endothelial NO or cGMP. This could be explained by differences in experimental conditions, *i.e.*, cell culture *versus ex vivo* studies, duration of exposure to L-glucose and, in diabetic animals, previous *in vivo* exposure to high glucose concentration as well as other metabolic abnormalities encountered in diabetes.

Both the endothelium (Zvgmunt *et al.*, 1995) and VSMC (Yeh *et al.*, 1996; Chaprie and Webb, 1993) contribute to NO production. Therefore, basal cGMP levels reflect the end product of endothelial and VSMC NO generation as well as other factors that may alter cGMP metabolism and guanylate cyclase activity. Although our data show that the high glucose buffer did not alter basal cGMP in vessels from control animals, it normalized levels in aortae from diabetic rats, an effect mimicked by the hyperosmolar effect of L-glucose. Since VSMC-derived NO may act in an autocrine manner to limit contractile activity (Yeh *et al.*, 1996; Chaprie and Webb, 1993), high basal cGMP levels in diabetic aortic rings in HG may be the result of a compensatory NO release from VSMC in response to high glucose-induced increases in vascular tone. Again, these data underscore the importance of the buffer glucose concentration in interpreting vascular NO production in the diabetic state.

In the vessels from diabetic rats, high glucose augmented contractile responses to KCl but not to NE while exposure to normal glucose lowered responses to both: normalizing responses to KCl and significantly attenuating sensitivity to NE. This may be explained by a glucose-mediated action on voltage-dependent $Ca^{2+}$ influx and/or mobilization of VSMC $[Ca^{2+}]_i$ from internal stores. A high glucose environment in VSMC has been reported to increase $Ca^{2+}$ influx (Barbagallo *et al.*, 1995), activate PKC by elevated diacylglycerol levels (Williams, 1995; Williams and Howard, 1994; Lee *et al.*, 1989; Inoguchi *et al.*, 1992) and inhibit $Na^+-K^+$ ATPase activity (Gupta *et al.*, 1992; Xia *et al.*, 1995). In addition, in cardiomyocytes, decreased cellular GSH was shown to lower $Na^+-K^+$ATPase activity (Haddock *et al.*, 1995). The data do not preclude the possibility of compensatory mechanisms that may mask abnormal PKC and $Na^+-K^+$ ATPase activity, including inhibition of PKC and activation of $Na^+-K^+$ pump activity by the enhanced endothelial, and probably VSMC, NO production (Minamino *et al.*, 1997; Nishizawa *et al.*, 1996; Gupta *et al.*, 1994). On the other hand, oxidation of the thiol group of L-type $Ca^{2+}$ channels activates $Ca^{2+}$ influx, at least in cardiomyocytes (Campbell *et al.*, 1996). Therefore, the increased oxidative stress seen in our study may explain why enhanced NO production prevented augmented responses to NE but not to KCl considering that KCl-induced contraction is mainly mediated by voltage-dependent $Ca^{2+}$ influx (Himpens *et al.*, 1988), and may also explain why results from cell culture experiments differ from *ex vivo* ones. High glucose-induced impairment of endothelial NO production has been proposed to decrease $Na^+-K^+$ ATPase (Gupta *et al.*, 1992) but the current data do not support this notion.

The effects of hyperosmolality on VSMC remain unclear and controversial (Barbagallo *et al.*, 1995; Williams and Howard 1994; Nielsen *et al.*, 1995; Soleimani *et al.*, 1995). However, in this investigation, in the tail artery and aorta, high glucose had no effect on normal vessels, suggesting that neither high glucose nor elevated osmolality play a role in these vascular beds in normal rats in the short term (2 hr). On the other hand, maintaining a hyperosmolar environment in vessels from diabetic rats resulted in contractile responses similar to those kept in a high D-glucose concentration. Thus, contributing factors in our short-term diabetic model, insulinopenia, and abnormal protein, lipid and glucose metabolism may play a permissive role and allow expression of the high glucose and hyperosmolar effects on vascular contractility.

Many vascular pathologic changes may impact on contractility/relaxation in long-term diabetic animals. In our animal model, blood pressure, heart rate and regional blood flow were not different from controls (data not shown). However, permanent changes in the arterial wall, associated with longer duration of diabetes mellitus, are unlikely to be responsible for the vascular abnormalities observed in these 7–10 days diabetic rats. Indeed, advanced glycation end products are not seen before 3 weeks of SZ-induced diabetes (Rumble *et al.*, 1997). Although collagen deposition has been reported after one week of SZ-induced diabetes (Rumble *et al.*, 1997), this is unlikely to mediate impaired vascular relaxation since endothelium-independent relaxation remains intact 8 weeks after SZ treatment (Tesfamariam *et al.*, 1990). On the other hand, rapid glycation dependent on reactive glucose metabolites in endothelial cells may contribute to the overall oxidative stress and vascular dysfunction seen in the short term.

In summary, our data indicate that: (1) endothelium-dependent relaxation is altered in the tail artery in the early stages of experimental diabetes; (2) aortic Ach-stimulated cGMP responses in diabetes are enhanced; and (3) some glucose effects on vascular reactivity are mediated by its osmotic properties; thus, in short-term diabetic animals, hyperglycemia-enhanced compensatory mechanisms to oxidative stress aimed at maintaining vascular function are evident, at least in the tail artery and aorta, while similar mechanisms are not activated by a 2 hr exposure of normal vessels to high glucose.

### *Acknowledgments*

This investigative work was supported by a VA Merit grant and an NIH grant RO1-HD-24497. Dr. Hamaty had been a recipient of the Brasza Diabetes Fellowship Award.

### *References*

Abiru, T., Watanabe, Y., Kamata, K. and Kasuya, Y. (1993) Changes in endothelium-dependent relaxation and levels of cyclic nucleotides in the perfused mesenteric arterial bed from streptozotocin-induced diabetic rats. *Life Sci.*, **53**(1), PL7–12.

Archibald, V., Cotter, M. A., Keegan, A. and Cameron, N. E. (1996) Contraction and relaxation of aortas from diabetic rats: effects of chronic anti-oxidant and aminoguanidine treatments. *Naunyn Schiedebergs Arch. Pharmacol.*, **353**(5), 584–591.

Avogaro, A., Piarulli, F., Valerio, A., Miola, M., Calveri, M., Pavan, P., Vicini, P., Cobelli, C., Tiengo, A., Calo, L. and Del Parto, S. (1997) Forearm nitric oxide balance, vascular relaxation, and glucose metabolism in NIDDM patients. *Diabetes*, **46**, 1040–1046.

Barbagallo, M., Slian, J., Pang, P. K. and Resnick, L. M. (1995) Glucose-induced alterations of cytosolic free calcium in cultured rat tail artery vascular smooth muscle cells. *J. Clin. Invest.*, **95**, 763–767.

Bohlen, H. G. and Lash, J. M. (1993) Topical hyperglycemia rapidly suppresses EDRF-mediated vasodilation of normal rat arterioles. *Am. J. Physiol.*, **265**, H219–H225.

Campbell, D. L., Stamler, J. S. and Strauss, H. C. (1996) Redox modulation of L-type calcium channels in ferret ventricular fnyocytes. Dual mechanism regulation by nitric oxide and S-nitrosothoils. *Journal of General Physiology*, **108**, 227–233.

Chaprie, J. R. and Webb, R. C. (1993) Vascular myocyte-derived nitric oxide is an autocrine that limits vasoconstriction. *Biochemical and Biophysical Research Communication*, **194**, 763–768.

Cipolla, M. J., Porter, J. M. and Osol, G. (1997) High glucose concentrations dilate cerebral arteries and diminish myogenic tone through an endothelial mechanism. *Stroke*, **28**, 405–411.

Durante, W., Sen, A. K. and Sunaliara, F. A. (1988) Impairment of endothelium-dependent relaxation in aortae from spontaneously diabetic rats. *Br. J. Pharmacol.*, **94**, 463–468.

Fukao, M., Hattori, Y., Kanno, M., Sakuma, I. and Kitabatake, A. (1997) Alterations in endothelum-dependent hyperpolarization and relaxation in mesenteric arteries from streptozotocin-induced diabetic rats. *Br. J. Pharmacology*, **121**(7), 1383–1391.

Graier, W. F., Wascher, T. C., Lackner, L., Toplack, H., Krejs, G. J. and Kukovetz, W. R. (1993) Exposure to elevated D-glucose concentrations modulates vascular endothelial cell vasodilatory response. *Diabetes*, **42**, 1497–1505.

Graier, W. F., Simecek, S., Kukovetz, W. R. and Kostner, G. M. (1996) High glucose induced changes in endothelial $Ca^{2+}$/EDRF signaling are due to generation of superoxide anions. *Diabetes*, **45**, 1386–1395.

Gupta, S., Sussman, I., McArthur, C. S., Torheim, K., Cohen, R. C. and Ruderman, N. B. (1992) Endothelium-dependent inhibition of $Na^+$–$K^+$ ATPase activity in rabbit aorta by hyperglycemia: possible role of endothelium-derived nitric oxide. *J. Clin. Invest.*, **90**, 727–732.

Gupta, S., McArthur, C., Grady, C. and Ruderman, N. B. (1994) Stimulation of vascular $Na^+$–$K^+$-ATPase activity by nitric oxide: a cGMP-independent effect. *American Journal of Physiology*, **266**(5 Pt 2), H2146–H2151.

Haddock, P. S., Woodward, B. and Hearse, D. J. (1995) Cardiac $Na^+$–$K^+$ ATPase activity and its relation to myocardial glutathione status: studies in the rat. *Journal of Molecular & Cellular Cardiology*, **27**, 1185–1194.

Himpens, B., Matthijs, G., Somlyo, A. V., Butler, T. M. and Somlyo, A. P. (1988) Cytoplasmic free calcium, myosin light chain phosphorylation, and force in phasic and tonic smooth muscle. *J. Gen. Physiol.*, **92**, 713–729.

Houben, A. J. H. M., Schaper, N. C. and Kruseman, C. A. N. (1993) Acute effects of local hyperglycemia on peripheral blood flow in man. *Diabetes Medicine*, **10**, 39–43.

Houben, A. J. H. M., Schaper, N. C., De Haan, C. H. A., Huvers, F. C., Slaaf, D. W., De Leeuw, P. W. and Kruesman, C. A. N. (1994) The effects of 7-hours local hyperglycemia on forearm macro and microcirculatory blood flow and vascular reactivity in healthy man. *Diabetalogia*, **37**, 750–756.

Ibu, J. O. and Short, A. H. (1986) Interactions between monosaccharides and disaccharides during uptake by the perfused liver of rat. *Quarterly Journal of Experimental Physiology*, **71**, 599–607.

Inoguchi, T., Battan, R., Handler, E., Sportsman, J. R., Heath, W. and King, G. L. (1992) Preferential elevation of protein kinase C isoform $\beta$II and diacylglycerol levels in the aorta and heart of diabetic rats: differential reversibility to glycemic control by islet cell transplantation. *Proc. Natl. Acad. Sci. USA*, **89**, 11059–11063.

Johnstone, M. T., Creager, S. J., Scales, K. M., Cusco, J. A., Lee, B. K. and Creager, M. A. (1993) Impaired endothelium-dependent vasodilation in patients with insulin-dependent diabetes mellitus. *Circulation*, **88**, 2510–2516.

Kamata, K., Miyata, N. and Kasuva, Y. (1989) Impairment of endothelium-dependent relaxation and changes in levels of cyclic GMP in aorta from streptozotocin-induced diabetic rats. *Br. J. Pharmacol.*, **97**, 614–618.

Kuwayama, H., Ecke, M., Gerisch, G. and Haastert, P. J. (1996) Protection against osmotic stress by cGMP mediated myosin phosphorylation. *Science*, **271**, 207–209.

Lee, T. S., Saltsman, K. A., Ohashi, H. and King, G. L. (1989) Activation of protein kinase C by elevation of glucose concentration: proposal for a mechanism in the development of diabetic vascular complications. *Proc. Natl. Acad. Sci. USA*, **86**, 5141–5145.

Minamino, T., Kitakaze, M., Node, K., Funaya, H. and Hori, M. (1997) Inhibition of nitric oxide synthesis increases adenosine production *via* extracellular pathway through activation of protein kinase C. *Circulation*, **96**, 1586–92.

Nielsen, H., Bonnema, S. J. and Flyvbjerg, A. (1995) Effects of diabetes, insulin treatment and osmolality on contractility of isolated rat resistance arteries. *Pharmacology & Toxicology*, **77**, 209–215.

Nishizawa, S., Yamamoto, S. and Umura, K. (1996) Interrelation between protein kinase C and nitric oxide in the development of vasospasm after subarachnoid hemmorrhage. *Neurological Research*, **18**, 89–95.

Oyama, M. (1996) cGMP accumulation induced by hypertonic stress in Dictyostelium discoideum. *Journal of Biological Chemistry*, **271**(10), 5574–5579.

Pieper, G. M., Langestroer, P. and Siebeneich, W. (1997) Diabetes-induced endothelial dysfunction in rat aorta: role of hydroxyl radicals. *Cardiovascular Research*, **34**, 145–156.

Popescu, L. M., Panoiu, C., Hinescu, M. and Nutu, O. (1985) The mechanism of cGMP-induced relaxation in vascular smooth muscle. *European Journal of Pharmacology*, **107**, 393–394.

Poston, L. and Taylor, P. D. (1995) Endothelium-mediated vascular function in insulin-dependent diabetes mellitus. *Clinical Science*, **88**, 245–255.

Rocha, G., Bucher, B., Tschopl, M. and Stoclet, J. C. (1995) Hyperosmolarity enhances smooth muscle contractile responses to phenylephrine and partially impairs nitric oxide production in the rat tail artery. *J. Vasc. Res.*, **32**(2), 119.

Rose, R. C. and Bode, A. M. (1995) Analysis of water-soluble antioxidants by high pressure liquid chromatography. *Biochemical Journal*, **306** (Pt. 1), 101–105.

Ruderman, N. B., Williamson, J. R. and Brownlee, M. (1992) Glucose and diabetic vascular disease. *FASEB J.*, **6**, 2905–2914.

Rumble, J. R., Cooper, M. E., Soulis, T., Cox, A., Wu, L., Yousef, S., Jasik, M., Gerums, G. and Gilbert, R. E. (1997) Vascular hypertrophy in experimental diabetes: role of advanced glycation products. *J. Clin. Invest.*, **99**, 1016–1027.

Soleimani, M., Singh, G., Dominguez, J. H. and Howard, R. L. (1995) Long-term hyperosmolality activates $Na^+$–$K^+$ exchange and protein kinase C in aortic smooth muscle cells. *Cir. Res.*, **76**, 530–535.

Taylor, P. D. and Poston, L. (1994) The effect of hyperglycemia on function of rat isolated mesenteric artery. *Br. J. Pharmacol.*, **223**, 801–808.

Tesfamariam, B., Brown, M. L., Deykin, D. and Cohen, R. A. (1990) Elevated glucose promotes generation of endothelium-derived vasoconstrictor, prostanoids in rabbit aorta. *J. Clin. Invest.*, **85**, 929–932.

Walsh, M. F., Barazi, M., Pete, G., Muniyappa, R., Dunbar, J. C. and Sowers, J. R. (1996) Insulin-like growth factor I diminishes *in vivo* and *in vitro* vascular contractility: role of vascular nitric oxide. *Endocrinology*, **137**, 1798–1803.

Wascher, T. C., Bachernegg, M., Kickenweiz, E., Stark, G., Stark, U., Toplak, H., Graier, W. F. and Krejs, G. S. (1995) Elevation of D-glucose impairs coronary artery autoregulation after slight reduction of coronary flow. *Europ. J. Clinical Investigation*, **25**, 590–594.

Williams, B. (1995) Glucose-induced vascular smooth muscle dysfunction: the role of protein kinase C. *Journal of Hypertension*, **13**, 477–486.

Williams, B. and Howard, R. L. (1994) Glucose-induced changes in $Na^+/H^+$ antiport activity and gene expression in cultured vascular smooth muscle cells: role of protein kinase C. *J. Clin. Invest.*, **93**, 2623–2631.

Xia, P., Kramer, R. M. and King, G. L. (1995) Identification of the mechanism for the inhibition of $Na^+$–$K^+$ adenosine triphosphate by hyperglycemia involving activation of protein C and cytosolic phospholipase $A_2$. *J. Clin. Invest.*, **96**, 733–740.

Yeh, J. L., Whitney, G., Lamb, S. and Brophy, C. M. (1996) Nitric oxide is an autocrine feedback inhibitor of vascular smooth muscle contraction. *Surgery*, **119**, 104–109.

Zvgmunt, P. M., Ryman, T. and Hogestatt, E. D. (1995) Regional differences in endothelium-dependent relaxation in the rat: contribution of nitric oxide and nitric oxide-independent mechanisms. *Acta Physiol. Scand.*, **144**, 257–266.

